# Celebrating Versatility: Febuxostat’s Multifaceted Therapeutic Application

**DOI:** 10.3390/life13112199

**Published:** 2023-11-11

**Authors:** Krasimir Iliev Kraev, Mariela Gencheva Geneva-Popova, Bozhidar Krasimirov Hristov, Petar Angelov Uchikov, Stanislava Dimitrova Popova-Belova, Maria Ilieva Kraeva, Yordanka Mincheva Basheva-Kraeva, Nina Staneva Stoyanova, Vesela Todorova Mitkova-Hristova

**Affiliations:** 1 Department of Propedeutics of Internal Diseases, Medical Faculty, Medical University of Plovdiv, 4000 Plovdiv, Bulgaria; 2 Second Department of Internal Diseases, Medical Faculty, Medical University of Plovdiv, 6000 Plovdiv, Bulgaria; 3 Department of Special Surgery, Medical Faculty, Medical University of Plovdiv, 6000 Plovdiv, Bulgaria; 4 Department of Otorhynolaryngology, Medical Faculty, Medical University of Plovdiv, 6000 Plovdiv, Bulgaria; 5 Department of Ophthalmology, Faculty of Medicine, Medical University of Plovdiv, 4000 Plovdiv, Bulgaria; 6 University Eye Clinic, University Hospital, 4000 Plovdiv, Bulgaria

**Keywords:** febuxostat, hyperuricemia, inflammation

## Abstract

Febuxostat, initially developed as a xanthine oxidase inhibitor to address hyperuricemia in gout patients, has evolved into a versatile therapeutic agent with multifaceted applications. This review provides a comprehensive overview of febuxostat’s mechanism of action, its effectiveness in gout management, its cardiovascular safety profile, renal and hepatic effects, musculoskeletal applications, safety considerations, and emerging research prospects. Febuxostat’s primary mechanism involves selective inhibition of xanthine oxidase, resulting in reduced uric acid production. Its pharmacokinetics require personalized dosing strategies based on individual characteristics. In gout management, febuxostat offers a compelling alternative, effectively lowering uric acid levels, relieving symptoms, and supporting long-term control, especially for patients intolerant to allopurinol. Recent studies have demonstrated its cardiovascular safety, and it exhibits minimal hepatotoxicity, making it suitable for those with liver comorbidities. Febuxostat’s potential nephroprotective effects and kidney stone prevention properties are noteworthy, particularly for gout patients with renal concerns. Beyond gout, its anti-inflammatory properties hint at applications in musculoskeletal conditions and a broader spectrum of clinical contexts, including metabolic syndrome. Emerging research explores febuxostat’s roles in cardiovascular health, neurological disorders, rheumatoid arthritis, and cancer therapy, driven by its anti-inflammatory and antioxidative properties. Future directions include personalized medicine, combination therapies, mechanistic insights, and ongoing long-term safety monitoring, collectively illuminating the promising landscape of febuxostat’s multifaceted therapeutic potential.

## 1. Introduction

Febuxostat, a xanthine oxidase inhibitor originally introduced for the management of hyperuricemia in patients with gout, has evolved into a multifaceted therapeutic agent with a spectrum of effects extending far beyond its initial scope [1]. This comprehensive review explores the diverse range of impacts exerted by febuxostat on various physiological systems, offering insights into its mechanism of action, efficacy in gout management, cardiovascular implications, renal and hepatic effects, musculoskeletal applications, adverse event profiles, and emerging avenues for future research.

Gout, characterized by the painful inflammation of joints due to the deposition of urate crystals, is intricately linked to elevated uric acid levels in the blood [1,2]. Febuxostat’s emergence as a promising treatment option stems from its pivotal role in lowering uric acid levels through the inhibition of xanthine oxidase, a key enzyme in uric acid production. However, as its clinical utilization has expanded, investigators have unearthed a wealth of additional effects and applications that broaden the therapeutic horizon of this medication [3].

Understanding the pharmacokinetics of febuxostat is paramount to optimizing its use. Its mechanism of action is underpinned by the selective and potent inhibition of xanthine oxidase, providing an effective means of curbing uric acid production [4,5]. Nevertheless, individual patient characteristics, such as age, sex, and renal function, necessitate careful consideration when determining appropriate dosing regimens.

Febuxostat’s efficacy in gout management is well established. It effectively reduces uric acid levels, provides relief from gout symptoms, and supports the long-term management of this debilitating condition. Its utilization is especially noteworthy for gout patients with contraindications to allopurinol, another common medication used for gout treatment [6,7].

Beyond its role in gout management, febuxostat has unveiled a broader spectrum of applications. Investigations into its cardiovascular effects have unveiled insights into its safety profile and its comparable cardiovascular impact to allopurinol. Moreover, the drug’s influence on hepatic function reveals its minimal hepatotoxicity, making it a viable option for gout patients with liver comorbidities [8,9].

Febuxostat’s effects extend into the realm of renal protection, potentially safeguarding against renal damage associated with hyperuricemia. Its role in preserving renal function, reducing uric acid levels, and preventing kidney stone formation has garnered attention [10,11].

The musculoskeletal arena has also seen the emergence of febuxostat as a potential candidate for applications beyond gout, including osteoarthritis management due to its anti-inflammatory properties [12].

As with any medication, a comprehensive understanding of febuxostat’s adverse event profile is crucial. Safety considerations have been a focal point of research, emphasizing the importance of vigilant monitoring, especially in specific patient populations.

Lastly, the burgeoning body of research exploring febuxostat’s potential impact on inflammation, cytokine regulation, and emerging avenues for its use in various inflammatory conditions underscores the drug’s expanding role in the realm of therapeutic interventions.

In summary, this review aims to provide a comprehensive exploration of the multifaceted effects of febuxostat, shedding light on its mechanism of action, efficacy in gout management, cardiovascular implications, renal and hepatic effects, musculoskeletal applications, safety profile, and emerging directions for further research. Febuxostat’s versatility as a therapeutic agent continues to unravel, offering new avenues for its utilization in diverse clinical contexts.

## 2. Febuxostat

**Figure 1:**
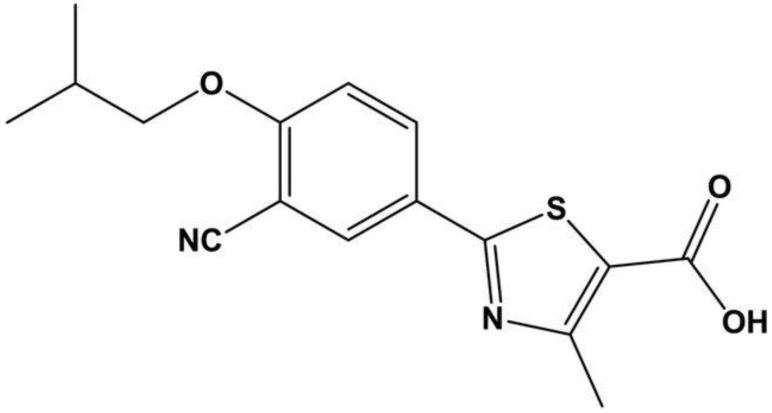
Chemical structure of febuxostat (Kaur, Manpreet et al., “Formulation and in vitro Evaluation of Fast Dissolving Tablets of Febuxostat Using Co-Processed Excipients” [13]).

Febuxostat is a xanthine oxidase inhibitor, a pharmaceutical agent employed in the management of hyperuricemia, particularly in the context of gout, a condition characterized by the deposition of uric acid crystals in joints (Figure 1). By selectively inhibiting the enzyme xanthine oxidase, febuxostat impedes the conversion of xanthine and hypoxanthine to uric acid, thereby reducing the production of uric acid in the body [1,2]. This mechanism of action helps to lower serum uric acid levels, mitigating the risk of acute gout flares and the formation of urate crystals in tissues. Febuxostat is considered a valuable pharmacotherapeutic option for patients with gout, hyperuricemia, or those susceptible to recurrent gout attacks, especially when conventional therapies prove inadequate or intolerable. Its clinical utility extends to individuals with conditions like renal impairment, where alternative medications may be less suitable [3,4].

### 2.1. Mechanism of Action and Pharmacokinetics

Febuxostat exerts its therapeutic effects through a well-defined mechanism of action and exhibits specific pharmacokinetic properties.

#### 2.1.1. Mechanism of Action

Febuxostat’s primary mechanism of action involves the inhibition of xanthine oxidase, an enzyme crucial in the production of uric acid. By inhibiting xanthine oxidase, febuxostat effectively reduces the conversion of xanthine and hypoxanthine into uric acid. This leads to decreased uric acid levels in the bloodstream, addressing the underlying cause of hyperuricemia, a condition characterized by elevated uric acid levels [4,5].

Febuxostat’s selectivity and potency in inhibiting xanthine oxidase contribute to its effectiveness in reducing uric acid levels. Unlike some older xanthine oxidase inhibitors, such as allopurinol, febuxostat does not rely on dose adjustments based on kidney function, making it a more straightforward option for many patients.

#### 2.1.2. Pharmacokinetics

Febuxostat’s pharmacokinetic properties vary with factors like age, sex, and renal function, necessitating consideration for dosing optimization. Some key pharmacokinetic aspects of febuxostat include:

- Absorption: febuxostat is well-absorbed after oral administration, with peak plasma concentrations typically reached within 1–1.5 h following ingestion [4].
- Distribution: The drug has a moderate volume of distribution, indicating its distribution throughout body tissues. It is bound to plasma proteins, primarily albumin [4].
- Metabolism: Febuxostat undergoes hepatic metabolism, primarily via the cytochrome P450 enzyme system. It is metabolized to inactive metabolites, which are subsequently excreted [5].
- Excretion: the drug and its metabolites are primarily excreted in the urine, with approximately 49% of the administered dose eliminated as unchanged febuxostat [4].
- Elimination Half-life: Febuxostat’s elimination half-life varies but is generally in the range of 5–8 h in healthy individuals. This property influences dosing frequency and considerations for patients with renal impairment [5].
- Dosing Considerations: Dosing adjustments may be necessary based on factors such as age and renal function. Patients with impaired renal function may require lower doses to prevent potential accumulation of the drug [5].

Understanding febuxostat’s pharmacokinetics is essential for healthcare providers to optimize dosing strategies for individual patients. It is particularly crucial to consider factors like age, sex, and renal function when prescribing febuxostat to ensure safe and effective treatment [4,5].

### 2.2. Efficacy in Gout Management

Febuxostat has proven efficacy in the management of gout, a painful inflammatory arthritis condition characterized by elevated uric acid levels in the blood [7]. By effectively reducing uric acid levels in the body, febuxostat provides relief from gout symptoms and supports long-term gout management [1,2]. Febuxostat’s effectiveness in reducing uric acid levels has been well-documented in clinical trials. It is considered an effective alternative for gout patients who may have contraindications to allopurinol, another common medication for gout [3].

Long-term gout management often involves the use of febuxostat as it supports sustained uric acid control. This is essential to preventing gout flares and the development of tophi, which are urate crystal deposits that can cause joint damage and deformities [3].

However, it is essential to recognize that gout management is not solely about reducing uric acid levels. Lifestyle modifications, dietary changes, and addressing comorbidities also play a crucial role in preventing gout flares and improving overall health.

### 2.3. Cardiovascular Effects and Safety Profile

Febuxostat has been the subject of an extensive investigation regarding its cardiovascular impact and safety profile [5].

Studies comparing febuxostat to allopurinol, another common medication for gout management, have indicated that febuxostat’s cardiovascular effects are comparable to those of allopurinol. This suggests that febuxostat does not pose a significantly higher cardiovascular risk compared to its counterpart. Furthermore, febuxostat demonstrates a favorable safety profile in various clinical settings. Reports of adverse events associated with febuxostat use are generally low, indicating that it is well-tolerated by many patients. Notably, febuxostat’s safety and tolerability have been confirmed in specific patient populations, including those with comorbidities such as hypertension or diabetes. This suggests that febuxostat may be a suitable choice for gout management in individuals with these common cardiovascular risk factors [14].

In the 2018 article “Cardiovascular Safety of Febuxostat or Allopurinol in Patients with Gout” by White et al., a randomized clinical trial assessed the cardiovascular safety of febuxostat and allopurinol in gout patients with cardiovascular risk factors. The study found that both medications had a similar risk of major cardiovascular events, indicating comparable cardiovascular safety. However, there was a suggestion of a potential increase in all-cause mortality with febuxostat, though this finding was not statistically significant. Gout flare rates did not significantly differ between the two treatment groups. The study’s results emphasize the need for individualized treatment decisions for gout in patients with cardiovascular comorbidities [15].

### 2.4. Renal Effects and Kidney Function

Febuxostat has garnered interest for its potential impact on renal function and its role in preventing kidney stone formation. Understanding these aspects is crucial for optimizing its clinical utility. Febuxostat exhibits potential nephroprotective effects, particularly in gout patients. It indicates that febuxostat may help prevent renal damage associated with hyperuricemia, which can lead to kidney complications. One significant advantage of febuxostat is its safe and effective use in gout patients with renal impairment.

By preserving renal function and reducing uric acid levels, febuxostat offers potential benefits in preventing kidney stone formation. Hyperuricemia is a known risk factor for kidney stone development, and febuxostat’s role in uric acid reduction may mitigate this risk [16].

A randomized, double-blind, placebo-controlled trial involving 467 patients with stage 3 chronic kidney disease (CKD) and asymptomatic hyperuricemia was conducted by Kimura et al. The goal was to determine if febuxostat, a urate-lowering therapy, could slow CKD progression. The primary outcome was the change in the estimated glomerular filtration rate (eGFR) over 108 weeks. The study found that febuxostat did not significantly impact eGFR decline in the overall group. However, subgroups without proteinuria and lower serum creatinine levels showed potential benefits. Notably, the febuxostat group had a lower incidence of gouty arthritis. In summary, febuxostat did not substantially slow CKD progression in stage 3 CKD patients with asymptomatic hyperuricemia, but some subgroups may benefit [17].

A study by Elsisi aimed to investigate the potential nephroprotective effects of febuxostat, mirtazapine, and their combination in countering gentamicin-induced kidney damage. Nephrotoxicity was induced in study subjects using gentamicin, and various treatments were administered before and during gentamicin exposure. The results demonstrated that both febuxostat and mirtazapine effectively mitigated the biochemical and histopathological changes induced by gentamicin. Moreover, they significantly reduced the renal levels of extracellular signal-regulated protein kinase 1/2 (ERK1/2) and monocyte chemoattractant protein-1 (MCP-1), which are associated with kidney injury. Importantly, the combination of febuxostat and mirtazapine showed a synergistic effect in protecting against gentamicin-induced nephrotoxicity. These findings suggest that nonpurine xanthine oxidase inhibitors like febuxostat, in combination with mirtazapine, may hold promise for future nephroprotective therapies. This novel approach offers potential opportunities for addressing kidney damage caused by nephrotoxic agents like gentamicin [18].

Febuxostat’s impact on kidney function appears to be favorable, but it is essential for healthcare providers to monitor renal parameters during treatment, particularly in patients with pre-existing kidney conditions. Regular kidney function tests can help assess the drug’s safety and effectiveness on an individual basis.

### 2.5. Liver Function and Hepatic Effects

Febuxostat is generally considered safe and well-tolerated by the liver. In clinical practice, it demonstrates minimal hepatotoxicity, making it a viable option for gout patients with underlying liver conditions. The hepatic effects of febuxostat are generally benign, and the drug does not exhibit significant hepatotoxicity, which is crucial for individuals with liver comorbidities who require uric acid-lowering therapy. Gout patients with liver conditions can benefit from febuxostat’s effectiveness in lowering uric acid levels without compromising liver function. This feature distinguishes it from other gout medications that may pose risks to the liver [5].

## 3. The Potential Impact of Febuxostat on Inflammation and Cytokines

Recent research on febuxostat has suggested that it may have additional benefits related to inflammation and cytokine regulation [8].

Several studies have explored the potential effects of febuxostat on inflammation and cytokine levels, shedding light on its broader therapeutic potential. Here, we delve into the findings of these studies to understand how febuxostat may impact inflammation and cytokine regulation.

### 3.1. Effects on Serum Cytokines

Febuxostat has sparked interest in its potential influence on serum cytokines such as IL-1, IL-4, IL-6, IL-8, TNF-α, and COX-2. A study conducted by Hao G, Duan W, Sun J, Liu J, and Peng B, and published in Experimental and Therapeutic Medicine in 2019 dived into this subject [8].

The study revealed intriguing findings about febuxostat’s effects on serum cytokines, that is, febuxostat appeared to influence the levels of various cytokines in the serum. These cytokines play pivotal roles in the body’s inflammatory responses and immune regulation. The study’s results hinted at febuxostat’s potential anti-inflammatory properties. By modulating proinflammatory cytokines like IL-1, IL-6, and TNF-α, febuxostat may contribute to dampening excessive inflammation [8]. Understanding febuxostat’s impact on serum cytokines has clinical significance, especially in conditions where inflammation plays a central role, such as gout and related arthritic conditions. While these findings are promising, more research is needed to elucidate the mechanisms through which febuxostat affects cytokine levels and its potential clinical applications. Clinical trials and studies in specific patient populations would provide valuable insights.

### 3.2. Heart failure, Aortic Fibrosis, and Inflammation

**Figure 2:**
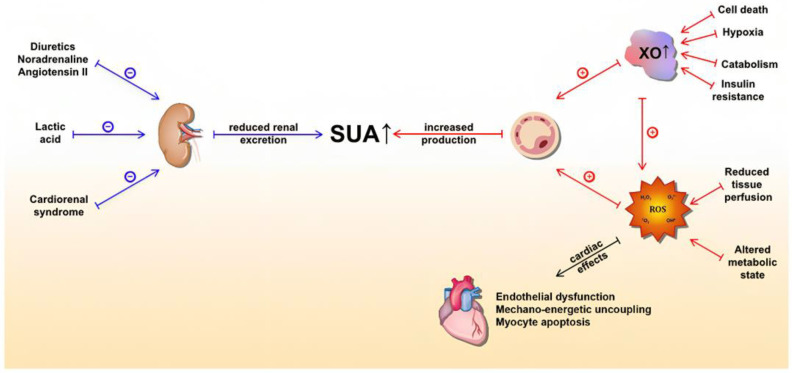
Underlying molecular mechanisms of serum uric acid (SUA) elevation in heart failure and detrimental effects of xanthine oxidase activity (mediated by ROS) on the heart. The blue lines represent mechanisms that lead to a reduction in UA excretion, whereas the red lines represent mechanisms that lead to increased UA production. Abbreviations: SUA: serum uric acid; XO: xanthine oxidase; ROS: reactive oxygen species. Kumrić, Marko et al., 2021. “Clinical Implications of Uric Acid in Heart Failure: A Comprehensive Review” [9].

Multiple large population studies indicate that UA is an independent predictor of mortality in acute and chronic HF, making it a significant prognostic factor in both settings. High serum levels have also been associated with an increased incidence of HF, thus expanding the clinical utility of UA. Importantly, emerging data suggests that UA is also implicated in the pathogenesis of HF, which sheds light on UA as a feasible therapeutic target (Figure 2).

This drug shows potential benefits in mitigating aortic fibrosis, a condition associated with inflammation and cardiovascular health. A study conducted by Kondo M et al. and published in the American Journal of Hypertension in 2019 explored the inhibition of xanthine oxidase by febuxostat in macrophages and its impact on aortic fibrosis induced by angiotensin II [10].

Several noteworthy findings emerged from this research:

- Febuxostat may have the ability to suppress aortic fibrosis, potentially due to its anti-inflammatory properties.
- Aortic fibrosis, often associated with inflammation, could be mitigated by febuxostat’s anti-inflammatory effects.

This suggests that febuxostat’s impact can potentially encompass cardiovascular health.

### 3.3. Gouty Arthritis and Gut Microbiota

Febuxostat has shown intriguing connections to gut microbiota and their potential influence on gouty arthritis. A study conducted by Lin X et al. and published in Frontiers in Pharmacology in 2020 explored these relationships [11].

The study examined the effects of a traditional Chinese medicine called Simiao Decoction, which contains febuxostat, on gouty arthritis and the gut ecosystem. Several key points emerge from this investigation: Febuxostat, as part of Simiao Decoction, exhibited the potential to alleviate gouty arthritis, a painful form of inflammatory arthritis, by modulating proinflammatory cytokines. This suggests a role for febuxostat in managing gout-related inflammation. The gut microbiota, composed of diverse microorganisms in the digestive tract, plays a vital role in regulating immune responses and inflammation. Alterations in gut microbiota composition have been linked to various inflammatory conditions, including gout [11].

Febuxostat’s potential influence on gut microbiota and its subsequent impact on gouty arthritis highlights the complex interplay between the drug, the gut ecosystem, and inflammation.

Understanding these relationships could offer insights into novel approaches for managing gouty arthritis by targeting gut microbiota composition and the associated inflammatory responses.

### 3.4. Ulcerative Colitis and Inflammation

In a study published in International Immunopharmacology in 2019, Amirshahrokhi K investigated the potential of febuxostat in mitigating ulcerative colitis in mice. The findings from this study suggest that febuxostat may exert anti-inflammatory effects in the context of ulcerative colitis [8].

Febuxostat’s anti-inflammatory effects in ulcerative colitis may be linked to its inhibition of the NF-κB signaling pathway, a key regulator of inflammation. This inhibition can reduce the production of proinflammatory cytokines, contributing to its anti-inflammatory properties [8].

### 3.5. Renal Injury and Signaling Pathways

Research conducted by Abdel-Wahab BA et al. in 2023, published in Biochemical Pharmacology, explored the protective effects of febuxostat against renal injury [16]. The study highlighted the crosstalk between signaling pathways involved in renal injury, including NLRP3/TLR4, Sirt-1/NF-κB, and TGF-β. Febuxostat’s potential involvement in modulating these pathways sheds light on its protective effects against inflammation and renal damage [18,19].

The intricate interplay between signaling pathways plays a pivotal role in renal injury. Febuxostat’s ability to influence these pathways suggests its potential to prevent or ameliorate renal damage associated with various insults. The NLRP3/TLR4 pathway is associated with inflammation and immune responses. Febuxostat’s modulation of this pathway indicates its potential anti-inflammatory effects in the context of renal injury. The Sirt-1/NF-κB pathway plays a role in oxidative stress and inflammation. Febuxostat’s involvement in this pathway suggests its ability to mitigate oxidative stress and inflammation in the kidneys.

Transforming growth factor-beta (TGF-β) signaling contributes to tissue fibrosis and scarring. Febuxostat’s potential to influence TGF-β signaling hints at its role in reducing renal fibrosis, a common consequence of kidney injury. Understanding febuxostat’s impact on these signaling pathways holds promise for renal protection. By targeting multiple pathways involved in renal injury and inflammation, febuxostat may offer a multifaceted approach to preserving kidney function [20,21].

A post hoc analysis of the febuxostat for Cerebral and Cardiovascular Events Prevention Study (FREED), which included 1070 asymptomatic, hyperuricemic elderly patients with cardiovascular risk factors, was conducted. These patients were divided into febuxostat and non-febuxostat groups, and the study assessed renal outcomes, including a 40% decline in estimated glomerular filtration rate, new-onset microalbuminuria, and the development or worsening of macroalbuminuria. The results showed that the febuxostat group had a 56% lower relative risk of developing or worsening macroalbuminuria, indicating a protective effect. However, the risks for other renal outcomes were similar. In asymptomatic hyperuricemic patients without gout, febuxostat was found to reduce the risk of macroalbuminuria development or worsening [22].

The potential application of febuxostat in preventing or attenuating renal injury is of clinical significance. Patients at risk of renal damage due to various factors, including exposure to nephrotoxic substances, may benefit from further research into febuxostat’s renal protective effects.

### 3.6. The Potential Impact of Febuxostat on Lung Inflammation

Some researchers have explored its potential to mitigate lung inflammation, particularly in the context of respiratory conditions. In a study published in Naunyn-Schmiedeberg’s Archives of Pharmacology in 2016, Fahmi AN et al. investigated the protective effects of febuxostat against lung inflammation induced by lipopolysaccharide in rats [23]. Their research suggested that febuxostat could mitigate lung inflammation in a dose-dependent manner, indicating its anti-inflammatory potential in lung-related conditions.

The observation that febuxostat may have anti-inflammatory effects on lung tissue extends its potential therapeutic applications beyond gout. Lung inflammation is a common feature of respiratory diseases, including chronic obstructive pulmonary disease (COPD) and acute respiratory distress syndrome (ARDS). If febuxostat can effectively reduce lung inflammation, it may offer a novel approach to managing these conditions [23].

The precise mechanisms through which febuxostat exerts its anti-inflammatory effects in the lungs require further investigation. Understanding these mechanisms is crucial for assessing the drug’s potential in treating lung-related inflammatory conditions.

While preclinical studies in animal models are promising, translating these findings to clinical applications in humans requires additional research. Clinical trials are necessary to evaluate the safety and efficacy of febuxostat in managing lung inflammation and its potential role in respiratory disease management.

### 3.7. The Role of Febuxostat in Modulating Platelet-Derived Microparticles (PMPs) and Adiponectin

As stated above, emerging research has suggested that febuxostat possesses anti-inflammatory properties, raising the question of whether it could influence PMPs and adiponectin levels as part of its anti-inflammatory mechanisms. Elevated PMP levels are associated with increased cardiovascular risk, and if febuxostat can effectively reduce PMP levels, it may indirectly contribute to cardiovascular risk reduction [24]. Adiponectin, known for its vasoprotective and anti-inflammatory effects, plays a crucial role in cardiovascular health. If febuxostat can positively influence adiponectin secretion or activity, it might offer additional cardiovascular protection beyond its primary function. The potential synergy between febuxostat, PMPs, and adiponectin is an area of interest. If febuxostat’s anti-inflammatory properties lead to a reduction in PMPs and an enhancement of adiponectin’s protective effects, it could have significant implications for cardiovascular disease prevention. Research on febuxostat’s effects on PMPs and adiponectin is still in its early stages, and clinical studies are needed to assess the impact of febuxostat on these factors, especially in individuals with hyperuricemia and gout who are at higher risk of cardiovascular disease. If febuxostat is found to positively influence PMPs and adiponectin, it could open doors to novel therapeutic strategies for managing cardiovascular disease and inflammation, potentially involving the use of febuxostat in specific patient populations or in combination with other cardiovascular medications. Continued investigation into febuxostat’s impact on PMPs and adiponectin is essential, including both preclinical studies to elucidate mechanisms and clinical trials to assess real-world effects and safety.

While the precise mechanisms and clinical implications are still being explored, these findings open intriguing avenues for future research and potential therapeutic applications [24].

### 3.8. Reduction of Synovitis by Febuxostat

Synovitis, the inflammation of the synovial membrane lining the joints, is a hallmark feature of many inflammatory arthritic conditions, including gouty arthritis [25].

The deposition of urate crystals in the joints can trigger an inflammatory response in the synovial membrane, leading to synovitis. By lowering uric acid levels, febuxostat indirectly mitigates this inflammatory cascade [25].

Clinical studies and observations have provided insights into the potential reduction of synovitis by febuxostat in gout patients. In some cases, patients receiving febuxostat therapy have reported improvements in joint pain, swelling, and tenderness, all of which are indicative of reduced synovitis. These observations align with the idea that lowering uric acid levels may help alleviate the inflammatory burden on the synovial membrane [25,26].

Febuxostat’s potential role in reducing synovitis has significant implications for gout management. It can complement existing treatments for gouty arthritis, such as nonsteroidal anti-inflammatory drugs (NSAIDs) and colchicine, by addressing the underlying uric acid-related inflammation. This combination therapy approach may lead to better symptom control and improved overall outcomes for gout patients.

Reducing synovitis is particularly crucial in the early stages of gouty arthritis. If synovitis is left untreated or inadequately managed, it can progress to more severe joint damage and chronic inflammation. Febuxostat’s ability to target the uric acid-related component of synovitis offers the potential for early intervention and prevention of disease progression.

## 4. Emerging Applications for Febuxostat

Recent studies have begun to unveil the broader therapeutic potential of febuxostat beyond its established roles. These emerging applications highlight the versatility of this medication and the promise it holds in various clinical contexts.

### 4.1. Cardiovascular Health

The cardiovascular implications of febuxostat extend beyond its primary function of managing hyperuricemia and gout. Notably, the medication has demonstrated a favorable cardiovascular safety profile, making it a compelling candidate for further exploration in the field of cardiac care [27].

Numerous studies have ignited interest in the possibility that febuxostat’s multifaceted properties could be harnessed to benefit individuals with various cardiovascular diseases [28]. One of the most intriguing aspects is its potential anti-inflammatory properties. Inflammation plays a pivotal role in the development and progression of conditions like atherosclerosis, heart failure, and other cardiac ailments [29]. Emerging research suggests that febuxostat may possess anti-inflammatory attributes that could offer significant advantages in the management of these cardiovascular disorders.

Atherosclerosis, characterized by the buildup of plaque in the arteries, is a prime example of a condition where inflammation is a key driver [30]. Some studies indicate that febuxostat’s anti-inflammatory properties may help mitigate the inflammatory processes associated with atherosclerosis. By doing so, it could contribute to the stabilization of arterial plaques and the prevention of cardiovascular events such as heart attacks and strokes.

Heart failure, another critical cardiovascular condition, is often accompanied by chronic inflammation [31,32]. The anti-inflammatory potential of febuxostat raises the possibility of managing inflammation in heart failure patients, potentially improving their overall cardiac function and quality of life.

As research in this area advances, future clinical trials are anticipated to provide a more comprehensive understanding of febuxostat’s cardiovascular impact [33]. These trials will play a pivotal role in elucidating whether the medication can be integrated into the existing therapeutic strategies for atherosclerosis, heart failure, and related cardiac conditions [34,35]. The potential for febuxostat to ameliorate inflammation and enhance cardiovascular health represents an exciting frontier in the ongoing pursuit of innovative approaches to cardiac care.

### 4.2. Neurological Disorders

In recent years, there has been mounting interest in unveiling the potential of febuxostat in the realm of neurological conditions [36]. This interest stems from the medication’s multifaceted properties, including its anti-inflammatory and antioxidative attributes, which have ignited optimism over its neuroprotective capabilities. While the bulk of this research remains in the preclinical phase, the preliminary findings are encouraging and warrant further investigation [37].

Preclinical studies, conducted primarily in animal models, have been instrumental in shedding light on febuxostat’s potential neuroprotective benefits. These studies have ventured into the exploration of its effects on various neurodegenerative diseases, with a particular focus on conditions such as Parkinson’s and Alzheimer’s. The results garnered from these investigations have sparked a surge of interest in advancing to clinical trials to assess the medication’s potential in humans [38].

Parkinson’s disease, a debilitating neurodegenerative disorder, has been a focal point of these preclinical investigations. Animal models of Parkinson’s have offered valuable insights, demonstrating that febuxostat’s anti-inflammatory properties could mitigate the inflammatory processes that contribute to the degeneration of dopaminergic neurons [35]. Additionally, its antioxidative effects may play a crucial role in reducing oxidative stress, a hallmark of Parkinson’s disease [39].

Similarly, research into Alzheimer’s disease has unveiled promising outcomes. The disease is characterized by the accumulation of amyloid-beta plaques and tau tangles in the brain, leading to cognitive decline. Studies in animal models suggest that febuxostat’s anti-inflammatory properties could potentially curtail the neuroinflammation associated with Alzheimer’s. Furthermore, its antioxidative capabilities may help combat the oxidative damage that plays a significant role in disease progression [37,40].

While preclinical studies offer a compelling foundation for further exploration, the translation of these findings to human clinical trials is the next critical step. Clinical trials will be pivotal in determining whether febuxostat can deliver on its neuroprotective potential and provide meaningful benefits to individuals affected by neurological conditions.

### 4.3. Metabolic Syndrome

Metabolic syndrome, a cluster of conditions including obesity, insulin resistance, and hypertension, represents a significant global health challenge. Some investigations indicate that febuxostat may help mitigate components of metabolic syndrome, possibly through its anti-inflammatory actions and effects on oxidative stress. This area of research holds promise for individuals at risk of metabolic syndrome-related complications [24,41].

A study by Nadwa et al. aimed to compare the effects of febuxostat and allopurinol on metabolic syndrome (MS)-related changes in a rat model. The researchers induced insulin resistance and MS in adult male rats through a high-fructose diet over 8 weeks. They assessed various parameters, including weight, blood pressure, serum biochemistry, and antioxidant enzyme activities. Both febuxostat and allopurinol treatments led to significant improvements, reducing body weight, blood pressure, blood glucose, insulin levels, and lipid profiles, and enhancing kidney function and endothelial integrity compared to untreated rats. Notably, febuxostat was more effective than allopurinol at normalizing fasting glucose, uric acid levels, as well as antioxidant enzyme activities. In conclusion, xanthine oxidase inhibitors, including febuxostat and allopurinol, were found to ameliorate MS-related effects, with febuxostat showing mild superiority in improving various metabolic parameters [17].

### 4.4. Rheumatoid Arthritis

Rheumatoid arthritis, an autoimmune inflammatory disorder, presents an intriguing arena for febuxostat exploration. Some studies suggest that it may complement existing rheumatoid arthritis treatments by targeting inflammatory pathways. Clinical trials evaluating its role in rheumatoid arthritis management are ongoing, with initial findings suggesting potential efficacy [42,43].

### 4.5. Cancer Therapy and Oxidative Stress

Emerging evidence suggests that febuxostat’s multifaceted properties extend beyond traditional medical applications, with potential implications in the field of cancer therapy [44]. Notably, febuxostat’s anti-inflammatory and antioxidative properties [45,46] have piqued the interest of researchers in the context of cancer treatment [47]. Early-stage investigations hint at its capacity to enhance the effectiveness of certain cancer treatments while simultaneously mitigating treatment-related inflammation, marking a promising development in the ongoing quest for more effective oncology solutions [48,49].

One study conducted by Fukui et al. [50] delves into the potential impact of febuxostat on oxidative stress in patients. This study encompassed 43 hyperuricemia outpatients, segregated into two groups: one group received febuxostat as a novel treatment, while the other switched from allopurinol to febuxostat. The research evaluated various parameters, including uric acid levels, creatinine levels, estimated glomerular filtration rate, and indicators of oxidative stress, specifically derivatives of reactive oxygen metabolites (d-ROMs) and biological antioxidant potential (BAP). Measurements were taken before and after treatment, offering insights into the medication’s effects.

The results of this study are particularly intriguing. Febuxostat exhibited a significant reduction in uric acid levels, which is essential in the context of hyperuricemia management. Additionally, it yielded a noteworthy reduction in d-ROMs, indicating its potential antioxidative properties. This reduction in oxidative stress markers may have broader implications, especially in conditions where oxidative stress plays a significant role, such as certain types of cancer.

The potential synergy between febuxostat and conventional cancer treatments opens up new avenues in the realm of oncology. By curbing inflammation and addressing oxidative stress [45], febuxostat could improve the overall efficacy of cancer therapies, minimize treatment-related complications, and enhance the quality of life for cancer patients [47]. However, it is crucial to note that this area of research is still in its early stages, and further investigations are warranted to fully understand the scope of febuxostat’s contribution to cancer treatment [47,51].

### 4.6. Influence of Gender on Uric Acid and Urate-Lowering Therapy

The influence of gender on uric acid and urate-lowering drugs becomes evident through the examination of gout patients with cardiovascular diseases. Cheng et al. reported that the use of febuxostat reveals an increased risk of heart failure (HF) hospitalization, particularly notable among women with gout and heightened cardiovascular risk. This connection highlights the nuanced interplay between sex, gout, and cardiovascular health [52]. 

Simultaneously, an analysis of clinical trials testing serum uric acid (SUA) lowering drugs reveals a concerning trend of declining enrollment of women over time. This trend varies among different drugs, indicating persistent underrepresentation of women across various classes of SUA-lowering medications. These insights underscore the imperative need to incorporate sex-specific considerations in both real-world settings and clinical trial designs. By doing so, we can achieve a more comprehensive understanding of the intricate dynamics surrounding the impact of uric acid and urate-lowering drugs, fostering equitable representation and empowering informed healthcare decisions [53].

## 5. Discussion

Febuxostat’s impact extends far beyond gout and gouty arthritis. This comprehensive review highlights the diverse effects of febuxostat on various physiological systems, shedding light on its mechanism of action, efficacy in gout management, cardiovascular implications, renal and hepatic effects, musculoskeletal applications, and the potential to modulate inflammation and cytokine regulation.

The mechanism of action of febuxostat centers on the selective and potent inhibition of xanthine oxidase, a key enzyme involved in uric acid production. Understanding the pharmacokinetics of this medication is essential for tailoring treatments to individual patient needs, as factors like age, sex, and renal function can influence its efficacy.

Efficacy in gout management is a hallmark of febuxostat. Not only does it effectively lower uric acid levels, providing relief from gout symptoms, but it also supports long-term management. This versatility is especially beneficial for gout patients with contraindications to allopurinol, broadening the spectrum of available treatments.

Comparative studies suggest that febuxostat’s cardiovascular effects are on par with allopurinol, alleviating concerns about heightened cardiovascular risks. The medication’s favorable safety profile, with low rates of adverse events reported, further underscores its suitability for diverse patient populations, even those with comorbidities.

In the realm of renal health, febuxostat reveals potential nephroprotective effects. It not only aids in preventing renal damage associated with hyperuricemia but also proves safe and effective for gout patients with compromised kidney function. This dual benefit preserves renal function and curtails the formation of kidney stones.

Febuxostat’s versatility extends beyond gout management. Its anti-inflammatory properties hint at potential applications in musculoskeletal conditions, including osteoarthritis. This broader clinical scope implies that febuxostat may hold promise in managing conditions linked to hyperuricemia and could even find utility in addressing metabolic syndrome, although further research is required to establish its full therapeutic range.

Moreover, emerging research suggests that febuxostat may play a role in modulating inflammation and cytokine regulation. Studies investigating its effects on serum cytokines, aortic fibrosis, gout-related inflammation, ulcerative colitis, renal injury, lung inflammation, platelet-derived microparticles, and adiponectin allude to its broader therapeutic potential in managing various inflammatory conditions.

## 6. Conclusions

In conclusion, febuxostat’s journey from a gout management drug to a multifunctional therapeutic agent is an exciting testament to the evolving landscape of medicine. While its effects on uric acid control are well-established, ongoing research reveals its potential across diverse domains, from inflammation modulation to neuroprotection and beyond. As we delve deeper into its mechanisms and applications, febuxostat offers promise as a cornerstone in the development of personalized, targeted therapies for a wide array of medical conditions. Patients and healthcare providers alike can look forward to a future enriched by the multifaceted effects of this remarkable medication.

## Data Availability

Not applicable.
